# Relationship Between Quality of Life and Depression in Pregnant Women

**DOI:** 10.5812/nms.8518

**Published:** 2013-06-27

**Authors:** Fatemeh Abbaszadeh, Mahboobe Kafaei Atrian, Negin Masoudi Alavi, Azam Bagheri, Zohreh Sadat, Zahra Karimian

**Affiliations:** 1 Department of Midwifery, Faculty of Nursing and Midwifery, Kashan University of Medical Sciences, Kashan, IR Iran; 2 Trauma Nursing Research Center, Kashan University of Medical Sciences, Kashan, IR Iran

**Keywords:** Quality of Life, Depression, Pregnant Women

## Abstract

**Background:**

Quality of life differs for different people in different situations and is related to one's self-satisfaction with life. Quality of life is affected by health status.

**Objectives:**

The current study examined the relationship between quality of life and depression in pregnant women in Kashan city.

**Patients and Methods:**

A Case - control study was performed on 112 depressed pregnant women (Case Group) and 353 Non-depressed pregnant women (Control Group) who referred to the prenatal health care centers of Kashan University of Medical Sciences .They completed Short Form 36 Health Survey (SF-36) to assess the quality of life and the Beck Depression Inventory to assess the level of depressive symptoms. T-test, chi-square and Pearson correlation coefficient statistical tests were used for data analysis.

**Results:**

The findings showed that there was an inverse relationship between quality of life and depression in pregnancy (P = 0.0001). Average scores in all eight domains of quality of life were significantly lower in depressed pregnant women compared to non- depressed women. The strongest relationship was observed between depression and vitality (r =-0.52, P = 0.0001), mental health (r = -0.50, P = 0.001) and social functioning (r =-0.38, P = 0.001).

**Conclusion:**

Depressed pregnant women had a lower quality of life. The proper management of depression during pregnancy can improve the quality of life in women. It is recommended that antenatal services integrate screening for depression into routine antenatal care.

## 1. Background

While pregnancy is a common event for reproductive-age women, surprisingly little has been published on the physical and emotional changes that typically occur during pregnancy (1). During the pregnancy period, dozens of biochemical, physiological and anatomical changes occur in women's body (2) . Such changes are beyond their control and are regarded as first changes that leave a woman physically and mentally vulnerable (3). However, for most women, pregnancy is considered as an evolutionary process but for successful coping, there is a need for physical, interpersonal and familial adjustments. Pregnant women often suffer from mental-health problems (4). 

For some women, pregnancy is stressful enough to be causing mental illness. The disease may be a recurrence or worsening of pre-existing mental disorder or the start of a new disorder (5). Studies show that depression is common in pregnancy and its prevalence is 4% to 29%. The prevalence has been reported to be changing from 7.4%, to 12.8% , and 12% in the first, second and third trimester respectively (6). Also, the prevalence of depression during pregnancy has been reported to be between 25% to 30.6% in Iran (7, 8). Sex, age, marital status, and social, economical and cultural factors may affect the prevalence of depression. Wyman believes that Quality of Life (QOL) is affected by health status (9). Also, depression is strongly related to the decline of individual's perceived health and functional status (10). Maternal age, gestational age, parity, and income level all affect the QOL in pregnancy. Then, measurement of QOL is important in care-planning for mothers and babies. The policymakers and community health care societies may benefit from the QOL information (11). Recent studies have shown that lower physical and social functioning in pregnancy is associated with increased risk of preterm delivery (12, 13). This may increase the number of prenatal care visits and recurrent fetal testing (14). Abbaszadeh et al. showed that pregnant women had lowest level of QOL in “functional limitations due to physical health problems” and “vitality” in normal pregnancy (15). Nicholson et al. found that the physical dimension of QOL was significantly lower in depressed pregnant women in early pregnancy (16). However, only a few studies are available about QOL and mental problems in pregnant women. These studies have limited generalizability, because most of analyses were conducted among white and middle-class samples (17) or among women in the first trimester of pregnancy (16). Also, a little data exist for racially and economically diverse samples (2).

## 2. Objectives

Because QOL is affected by the beliefs and culture and due to the correlation between prenatal depression and physical or mental health among Iranian women was not previously investigated. The current research aimed to explore the relationship between depression and QOL in pregnant women in Kashan, Iran.

## 3. Patients and Methods

A Case-control study was performed on 112 depressed pregnant women (Case Group) and 353 Non-depressed pregnant women (Control Group) who referred to the prenatal health care centers of Kashan University of Medical Sciences from September to December 2006. Women were eligible to participate in this study if they could read and write and had gestational age 7-41 weeks. Also, not having a known mental illness or important medical or obstetrical problems resulting in a high-risk pregnancy, or a history of traumatic events such as the dead of close relatives and changing the job in the previous year were selected as additional inclusion criteria. All women were free to participate and were assured of confidentiality of their personal information.

Cluster random sampling was done. All the health centers in Kashan city were divided into five regions, and then two centers from every region were randomly selected. All women referring to these centers for prenatal care were recruited to the study. After explanation of the research objectives and obtaining informed consent, the women completed the depression and the SF-36 questionnaires.

Depression was assessed using the Beck Depression Inventory, second edition (BDI). BDI contains 21 multiple-choice self-report items that assess the intensity of depressive symptoms in areas such as mood, pessimism, guilt, sense of failure, suicidal thoughts, fatigue and weight loss. One can get the score from 0 to 63. A score higher than 16 is an indicator for depression (17). BDI is a valid and reliable questionnaire with 0.93 alpha-cronbach coefficient (7), and its Persian version has been used in different studies in Iran (18, 19).

SF-36 questionnaire was another tool that was used in the current study. This questionnaire is a general measure of patients’ health that can adequately assess the QOL. SF-36 questionnaire has 36 items in eight dimensions of physical functioning (10 items), social functioning (2 items), physical problems (4 items), emotional problems (3 items), mental health (5 items), vitality (4 items), pain (2 items) and general health perception (5 items). The potential score is from 0 to 100. A higher score shows a better health condition. The validity and reliability of the Persian version of this questionnaire have been already studied in Iran (alpha-cronbach between 0.77-0.9) (20).

After completing BDI by pregnant women, 112 women were placed in the case group (with score higher than 16) and 353 women in the control group (with scores equal to or lower than 16). The case and control groups were matched for maternal age, gestational age, parity, economic status, unwanted pregnancy and support of the spouse and statistical analysis (t-test and Chi-square) showed no significant difference between the two groups with respect to these variables.

Statistical analysis of the data was performed using SPSS version 11.5. Descriptive analyses were used to explore frequencies, means, and standard deviations to summarize data. Differences in means were analyzed using independent sample t-test. Chi-square test and Fisher's exact test were also applied to nominal variables. The relationships of QOL and depression were evaluated using a Pearson Correlation coefficient. Also, Odds ratio and 95% confidence interval were calculated. A P-value of less than 0.05 was regarded to be significant.

## 4. Results

In the current study, the majority of depressed and non-depressed subjects were aged between 18 - 23 years. The mean age was 25.4 ± 4.63 year and 25.32 ± 4.41 year in depressed and non-depressed women respectively. Also, the mean of gestational age was 26.69 ± 9.71 months and 24.93 ± 9.2 months in depressed and non-depressed women. Most of the subjects (36.7%) had a high school education (Table 1). The majorities of women were housewives (94.3%) and experienced their first pregnancy (57.4%).

**Table 1.** Comparison of Maternal Age, Gestational age and Parity in Depressed and Non-Depressed Women

Characteristics, Mean ± SD	Depressed, (n =353)	Non-depressed , (n =112)	95% CI^a^	P value	T -Test
**Maternal age, year**	25.40 ± 4.63	25.32 ± 4.41	-1.03-0.88	P = 0.66	0.16
**Gestational age, week**	26.69 ± 9.71	24.93 ± 9.20	-3.79-0.28	P = 0.08	-1.70
**Parity**	1.54 ± 0.74	1.66 ± 0.89	-0.24-0.85	P = 0.372	0.92

^a^ CI: Confidence Interval

**Table 2.** The Distribution of Income, Support of the Spouse and Unwanted Pregnancy in the Two Groups

Characteristics, No. (%)	Depressed, (n =353)	Non-depressed , (n =112)	P value	Chi-square	Odds Ratio
**Income**					
low	148 (41.9)	33 (29.5)	0.12	0.018	1.73
Moderate-high	205 (58.1)	79 (70.5)			
**Support of the spouse**					
Yes	311 (88.1)	94 (83.9)	0.129	0.251	1.10
No	42 (11.9)	18 (16.1)			
**Unwanted pregnancy**					
Yes	19 (17)	43 (12.2)	0.143	0.194	0.68
No	93 (83)	310 (87.8)			

The mean score of BDI was 11.79 ± 8.10 in women. Eighty women (17%) had moderate depression with the score of 17-24, and 32 women (7%) had severe depression with the score more than 24. Table 3 shows that means of QOL was lower in depressed women than non-depressed ones and that the depressed women had significantly lower scores in all dimensions, except "functional limitations due to physical health problems " compared to non-depressed ones.

**Table 3.** Comparison of Mean and Standard Deviation of Overall QOL and Its Dimensions in Depressed and Non-Depressed Groups

Dimensions of Quality of Life	Depressed, 16 <, (Mean ± SD)	Non-depressed, 16≤, (Mean ± SD)	95% Confidence Interval of the Difference	P value
**Overall quality of life**	52.85 ± 11.80	63.72 ± 12.86	5.96 - 11.11	0.001
**General health**	54.17 ± 15.67	64.09 ± 14.08	1.72 - 7.67	0.001
**Physical functioning**	57.10 ± 19.81	64.45 ± 22.42	1.26 - 10.81	0.007
**Functional limitations due to physical health problems**	53.36 ± 15.74	57.00 ± 19.35	4.15 - 12.22	0.073
**Functional ** **limitations due to emotional problems**	58.75 ± 19.99	65.78 ± 22.35	7.85 - 17.11	0.003
**Social functioning**	51.98 ± 21.87	67.76 ± 21.57	2.55 - 11.63	0.001
**Bodily pain**	50.09 ± 25.48	61.17 ± 24.77	3.40 - 13.86	0.001
**Vitality**	43.45 ± 16.38	59.72 ± 17.28	7.60 - 14.18	0.001
**Mental health**	52.45 ± 19.08	70.04 ± 16.25	7.32 - 14.31	0.001

Table 4 shows that depression was negatively correlated with all QOL subscale scores.

**Table 4.** The Association Between Depression and Dimensions of Quality of Life Groups in Pregnant Women

Dimensions of Quality of Life	Correlation coefficient	P Value
**General health**	- 0.37	0.001
**Physical functioning**	- 0.217	0.001
**Functional limitations due to physical health problems**	- 0.20	0.001
**Functional limitations due to emotional problems**	- 0.20	0.001
**Social functioning**	- 0.38	0.001
**Bodily pain**	- 0.29	0.001

## 5. Discussion

The results showed that QOL in depressed pregnant women was lower than that of non-depressed women. Average scores in all dimensions of QOL except "functional limitations due to physical health problems" were significantly lower in depressed pregnant women. Previous studies emphasize the importance of health-related QOL within the broader context of maternal health and pregnancy outcomes. Lower physical and social functioning in pregnancy has been linked to an increased risk of preterm birth (12, 13). Poor emotional functioning has been associated with an increase in primary care visits and resource use (21). In particular, poor maternal physical or emotional functioning could lead to an increase in prenatal visits or fetal testing (14).

Results of the present study were similar to those of Forozandeh et al. who found that depressed pregnant women had more health-related physical and functional impairment (22). Lee et al. also showed that depressed pregnant women suffer from impaired physical and mental health and lower levels of QOL (23). Another study have also reported that there is a negative correlation between QOL and depression in pregnant women with high risk pregnancies and that mean scores of QOL in pregnant women with moderate depression was significantly lower than that of pregnant women with no or mild depression (24). Couto et al. in a study on women with histories of recurrent abortion, fetal death and early neonatal death found an inverse correlation between the SF-36 domains and depression and that they had lower scores for physical functioning and higher scores in the domain of bodily pain than women without such histories (25). However the current study showed poorer QOL in depressed pregnant women than in non-depressed ones even in normal pregnancy.

Maki et al. found a negative relationship between QOL or and functional status and depression among low-income minority women in late pregnancy. They came to the conclusion that depressed pregnant women had lower scores in all eight domains of quality of life compared to non-depressed women. In another study, Nicholson et al. also found similar results in early pregnancy (16). This study suggests that depression may affect on all dimensions of QOL throughout pregnancy. Depression can also affect negatively on functions of the immune system and neuroendocrine pathways (26, 27)

The current study showed that quality of life scores in all dimensions were considerably lower in depressed women. Mental health score was 17 points lower in depressed women while the vitality was 16.27 points lower in depressed women. Nicholson et al. in their research in USA (2006) had the same results. They also found that depressed women had significantly lower scores on all dimensions of quality of life except the physical functioning. The score of pain was 19 points lower, and the score of physical problems was 30 points lower (14). It seems that the difference in quality of life scores in the two studies is due to the difference in subjects. In the current study, the pregnant women were healthy but in the Nicholson’s research the women with medical problems had also been recruited.

The current study broadens the understanding of the effect of depressive symptoms on health-related quality of life through pregnancy. This work signifies the importance of identifying depressive symptoms in pregnancy, the independent association of depressive symptoms with health-related QOL , and potential opportunities for physician training and intervention. Further studies are needed to determine whether the assessment and treatment of depressive symptoms in pregnant women can increase their quality of life. Future studies might also prospectively evaluate the effect of depressive symptoms on biologic mechanisms of pregnancy and perinatal outcomes.

The results of the current study showed that quality of life scores are lower in depressed pregnant women and depressed women suffer from impaired physical and mental health. Given the prevalence and severity of depression in healthy pregnant women, there is a need for depression screening by standard tests in the first visits in prenatal care. The proper management of depression during pregnancy can improve the QOL in the women. It is recommended that antenatal services integrate screening for depression into routine antenatal care.
